# Formation mechanism of Mn_*x*_Co_3−*x*_O_4_ yolk–shell structures†

**DOI:** 10.1039/d1ra04996h

**Published:** 2021-09-01

**Authors:** Chang-Yang Chiang, Wuzong Zhou

**Affiliations:** EaStChem, School of Chemistry, University of St Andrews Fife KY16 9ST UK wzhou@st-andrews.ac.uk

## Abstract

Formation of Mn_*x*_Co_3−*x*_O_4_ yolk–shell microspheres *via* a solvothermal reaction of hydrated cobalt and manganese nitrates in ethanol is investigated. Spinel nanocrystals of cobalt oxide or cobalt-rich ternary oxide preferentially develop in the system, while manganese-rich hydroxide form Mn(OH)_2_-type nanosheets. Instead of continuing to grow individually, the nanocrystallites and nanosheets aggregate into large microspheres due to their strong inter-particle interaction. When the proportion of Mn-rich nanosheets is high, therefore the overall density is low, dehydration of hydroxide nanosheets and a surface re-crystallisation lead to formation of a dense and rigid shell, which is separated from a solid or hollow core *via* a further Ostwald ripening process. The proposed formation mechanism of the yolk–shell structures based on electron microscopic studies would help us to develop yolk–shell structure based multifunctional materials.

## Abstract 2

A formation mechanism of yolk–shell microspheres of Mn-rich spinel Mn_*x*_Co_3−*x*_O_4_ is proposed based on the analyses of microstructures and local compositions.

## Graphical abstract

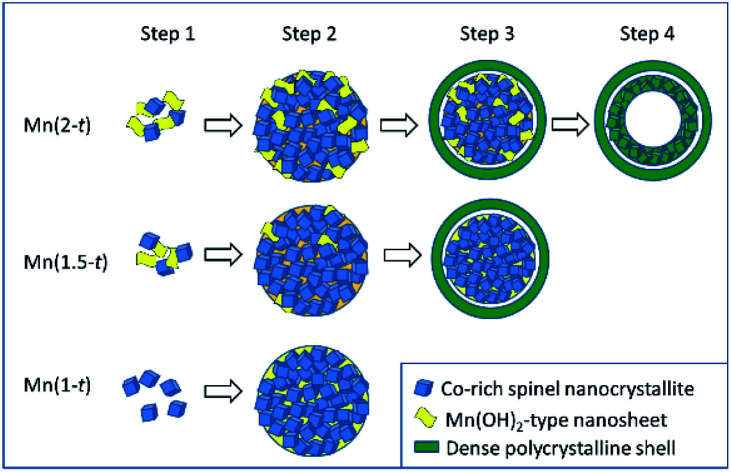

## Introduction

A large number of functional materials with spinel (AB_2_O_4_) structures have increasingly attracted researchers in various fields. One of the challenging targets in research of the spinel materials is controlling the particle size and morphology,^1–3^ in order to meet the requirements of different applications, *e.g*. in catalysis,^4–8^ Li recovery,^9^ energy storage^10^ and magnetic devices.^11^

In the application of catalysis, large crystals normally have low catalytic activity, while nanocrystallites suffer from self-aggregation or unexpected side reactions. Hierarchical constructions using nanoparticles into some special morphologies may overcome these shortcomings, *e.g*. porous nanorods,^12^ nanofibres,^13^ micro-cubes,^14^ micro-dumbbells^15^ and microspheres.^16–19^ These materials show several advantages, such as high morphological stability against the volume change, high specific surface areas for chemical reactions or porous structures for gas or liquid diffusion. Development and refinement of the synthesis methods for these spinel materials are therefore crucial.

Fu *et al.* presented an attractive solvothermal method for the synthesis of MnCo_2_O_4_ microspheres *via* aggregation of nanocrystals.^20^ In this one-step method, they used a relatively low reaction temperature without further calcination. It was also environmentally friendly since no surfactants or other organic compounds were needed except two metal nitrates as the precursors and ethanol as the solvent. It was found a redox reaction between ethanol and nitrate anions in the solution along with the formation of NO and N_2_O gases could play an important role in the formation of MnCo_2_O_4_ nanoparticles, that underwent self-assembly to the mesoporous microspheres. Fu *et al.* also observed yolk–shell microspheres only in a Mn-rich composition, Mn_2_CoO_4_. However, the detailed structures and formation mechanism of this hierarchical construction were not discussed.

In the present work, Mn_*x*_Co_3−*x*_O_4_ microspheres were prepared using a similar method as reported by Fu *et al.*^20^ It was found that, with an increase of the Mn content, the particle morphology changed from shell-free spheres to solid yolk–shell spheres and finally to hollow yolk–shell spheres. Unlike multi-step or templating syntheses of many other core–shell structures,^21–23^ the formation of yolk–shell Mn-rich spinel microspheres in one-step solvothermal synthesis has several advantages as mentioned above and the formation mechanism is proposed based on the local structural analysis using electron microscopy. The solid yolk–shell structure has a solid core covered by a shell with a gap between them. The hollow yolk–shell structure contains a hollow core. Both have a large inner surface and space. The diffusion length of ions in these materials can be greatly reduced. The compositions of the “yolk” and the shell may be different. This work may shed light on the development of yolk–shell functional materials for potential applications in various fields, *e.g.* catalysis, drug delivery, nanoreactors, lithium-ion batteries, *etc.*^24^

## Results and discussion

### Structures of specimens after reaction for 24 h

**Fig. 1:**
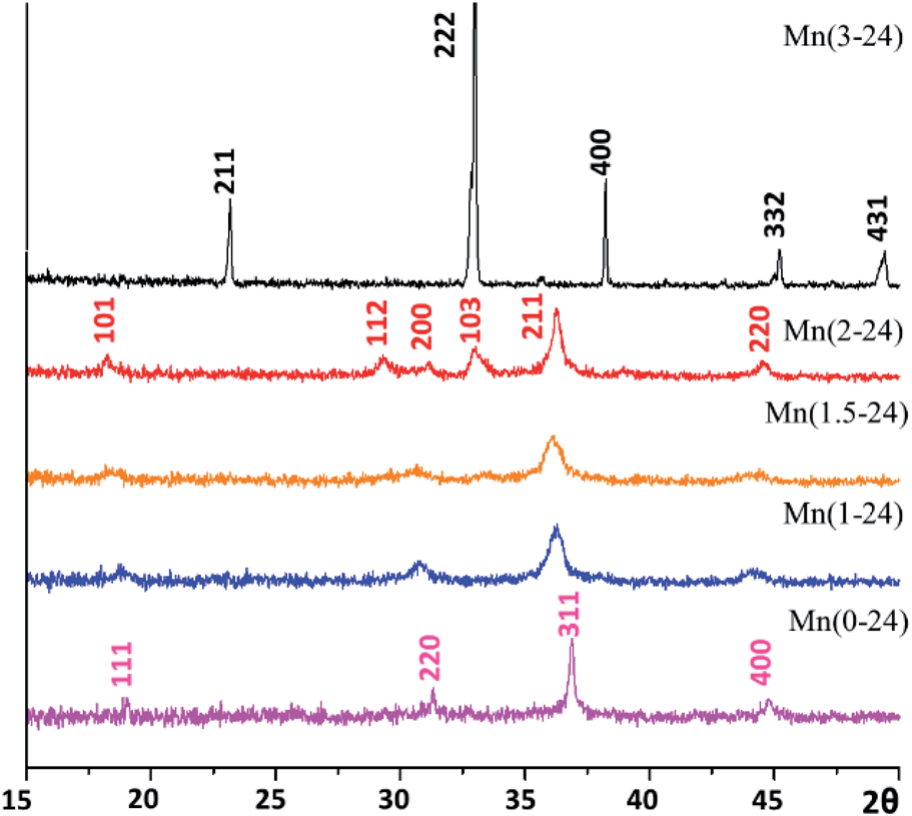
PXRD patterns recorded from the Mn(*x*-24) specimens. The pattern of Mn(3-24) is indexed to the body-centred Mn_2_O_3_ structure. The pattern of Mn(0-24) is indexed to the face-centred cubic spinel structure of Co_3_O_4_. The pattern of Mn(2-24) is indexed to the tetragonal spinel structure of Mn_2_CoO_4_.

The crystalline phases of the samples were initially detected by powder X-ray diffraction (PXRD). As shown in Fig. 1, all the samples prepared with a reaction time of 24 h with different nominal ratios of Mn/Co look like monophasic. The produced specimens were designated as Mn(*x-t*), where *x* is the Mn content in the nominal formula Mn_*x*_Co_3−*x*_O_4_, and *t* is reaction time in hours. The PXRD pattern of Co-free Mn(3-24) shows sharp peaks indicating bulk crystals and can be indexed to the cubic Mn_2_O_3_ structure (JCPDS card no. 04-007-0856) with the unit cell dimension, *a* = 9.5 Å, space group *Ia*3. In the crystal growth, all the Mn^2+^ cations were oxidised to Mn^3+^. All other PXRD patterns show relatively weak and wide diffraction peaks, implying that the samples contain nanocrystallites. The pattern of the Mn-free Mn(0-24) specimen is indexed to pure cubic spinel structure of Co_3_O_4_ (JCPDS card no. 04-016-4508), with the calculated unit cell parameter of *a* = 8.0756 Å, space group *Fd*3̄*m*.

Equivalent Mn and Co cations have similar ionic radii. Therefore, Mn can easily replace Co in the spinel structure to form solid solutions. On the other hand, since Mn cations are slightly larger than Co cations, with an increase of Mn-doping in Co_3_O_4_, the cubic structure undergoes a tetragonal distortion. The PXRD pattern of Mn(2-24) is indexed to a tetragonal spinel structure with the calculated unit cell parameters of *a* = 5.737 Å and *c* = 9.2391 Å, which are close to the reported cell dimensions of Mn_2_CoO_4_ (JCPDS card no. 04-001-7706). The PXRD patterns of Mn(1.5-24) and Mn(1-24) can be regarded as intermediate phases in the phase transformation between cubic Co_3_O_4_ and tetragonal Mn_2_CoO_4_.

The particle size and morphology of the produced samples were revealed by scanning electron microscopy (SEM) and transmission electron microscopy (TEM) images. As it can be expected after the PXRD study, the Co-free Mn(3-24) are relatively large octahedral crystals. Many particles are clusters of intergrown small octahedra, that could be resulted from multiple nucleation at each precursor particle (Fig. S1, ESI†).

**Fig. 2:**
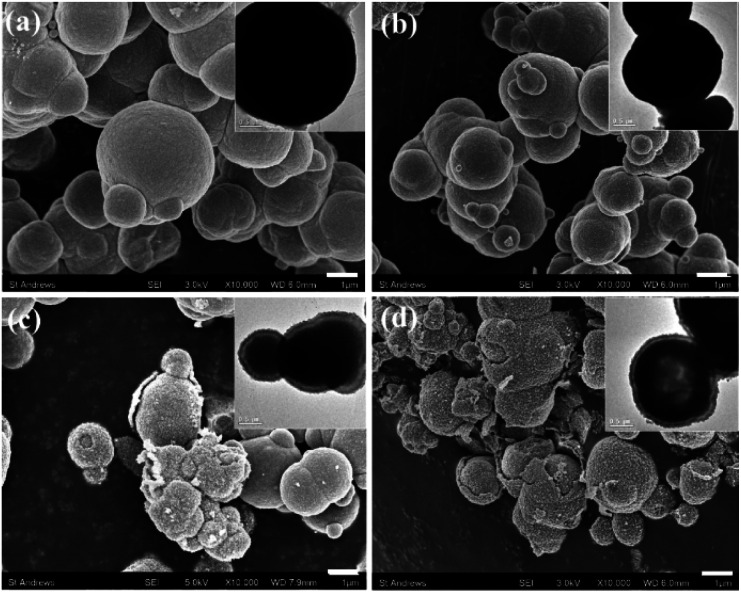
Typical SEM images and the corresponding TEM images from (a) Mn(0-24), (b) Mn(1-24), (c) Mn(1.5-24) and (d) Mn(2-24). The scale bars are 1 μm.

The particle morphologies of all other Co-containing samples are spherical. Most of the particles of Mn-free Mn(0-24) specimen are dense microspheres with a diameter of 1 to 4 μm (Fig. 2a). High resolution SEM images show that these microspheres are polycrystalline, containing self-assembled nanoparticles (Fig. S2, ESI†). The formation of polycrystalline microspheres is a crucial step in the so-called reversed crystal growth of some zeolites.^25^ The surface of the spherical aggregates became the most active site for crystallisation and may re-crystallise into single crystal polyhedral shells. Such surface re-crystallisation into single crystal shell did not take place in the (Mn, Co)_3_O_4_ microspheres in the present work. The re-crystallisation was probably suppressed by the organic solvent.

With Mn-doping, the produced particles of Mn(1-24) are also dense microspheres (Fig. 2b). When the Mn content further increases, the particles in Mn(1.5-24) seem to have a core–shell structure (Fig. 2c). Each particle has a thin shell and a solid core. A gap between the shell and the core can be seen in the SEM images when shell is broken or in the TEM images where the gap shows a light contrast according to the mass-thickness contrast formation mechanism. Therefore, these microspheres have a yolk–shell structure. When the Mn content increases to be higher than Co, many microspheres in Mn(2-24) show an hollow core as seen in the inset of Fig. 2d. The solid core and hollow core can be confirmed by SEM images when the cores are broken (Fig. S3, ESI†).

Alongside the morphological change, the SEM images in Fig. 2 also show that the surface roughness of the microspheres increase with the increase of the manganese content. To understand the reason for the difference of surface roughness, high resolution TEM (HRTEM) images were recorded to detect the individual nanocrystallites.

**Fig. 3:**
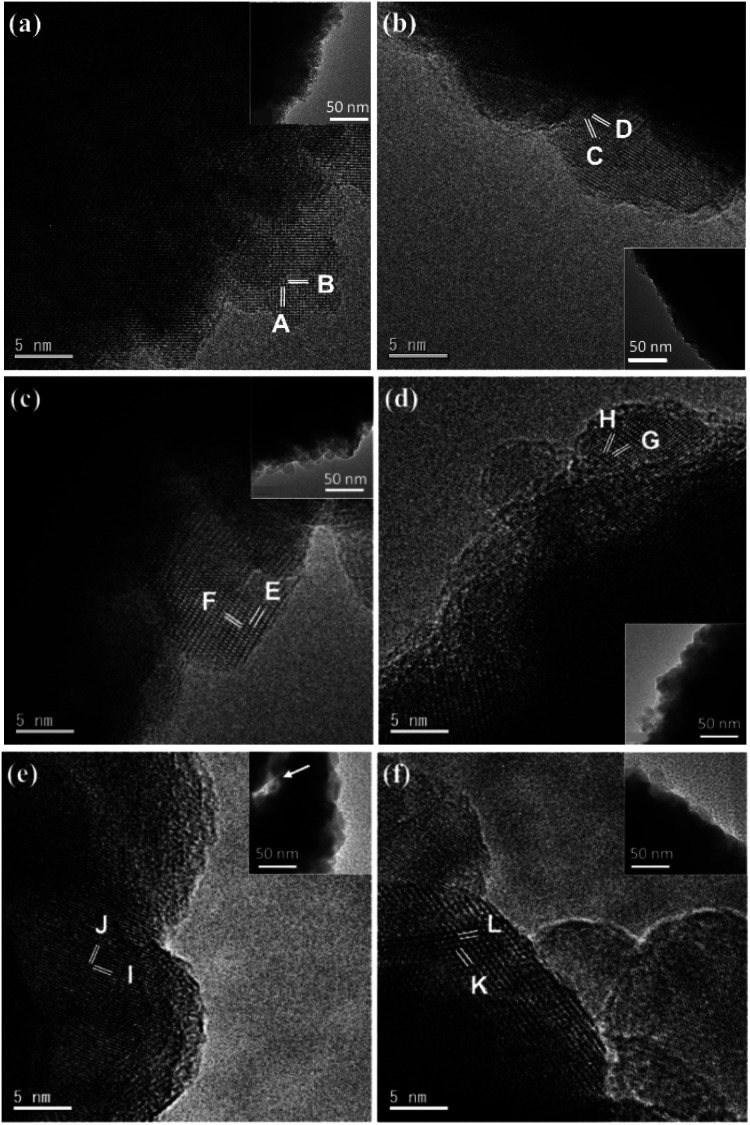
HRTEM images of spinel microspheres with the corresponding TEM images in the insets. The scale bars are 5 nm. (a) Surface image from a microsphere in Mn(0-24). The fringes are indexed to the cubic Co_3_O_4_, A: (22̄0)_C_ and B: (222)_C_. (b) Surface image from a microsphere in Mn(1-24). The fringes are indexed to a pseudo-cubic spinel structure, C: (31̄1̄)_C_ and D: (31̄1)_C_. (c and d) Images of a yolk–shell microsphere in Mn(1.5-24) at the (c) shell and (d) core surfaces. The lattice fringes are indexed to tetragonal spinel unit cells, E: (101)_T_, F: (11̄2̄)_T_, G: (013)_T_, H: (103)_T_. (e and f) Images of a hollow yolk–shell microsphere in Mn(2-24) at the (e) shell and (f) core surfaces. The lattice fringes are indexed to tetragonal unit cells, I: (103)_T_, J: (2̄02)_T_, K: (011)_T_, L: (011̄)_T_. The arrow in the inset of (e) indicates a gap between the shell and the core.

Unlike the single crystal property of the octahedral particles of Mn_2_O_3_ in Mn(3-24) (Fig. S1, ESI†), all the microspheres with different compositions are polycrystalline. Fig. 3a is a surface HRTEM image of a microsphere in Mn(0-24), revealing many partly orientated ultra-small crystallites of <5 nm in diameter. The crystal size in the inner area is slightly larger. The measured lattice fringes, *d*_A_ = 2.85 Å and *d*_B_ = 2.33 Å, can be indexed to the (22̄0) and (222) planes of the cubic spinel structure of Co_3_O_4_. The ultra-small particle size explains the relatively smooth surface in comparison with the microspheres in other compositions with the Mn-doping. The partial ordering of the nanoparticles also implies some degree of intergrowth of the nanocrystallites. Consequently, the PXRD peaks of Mn(0-24) are relatively sharper than the peaks from other spinel specimens as shown in Fig. 1.

The crystal size at the microsphere surface from sample Mn(1-24) is larger than that of Co_3_O_4_ (Fig. 3b). The measured *d*-spacings are *d*_C_ = 2.43 Å and *d*_D_ = 2.47 Å with an interplane angle of 35.5°, which are indexed to the (31̄1̄) and (31̄1) planes of a pseudo-cubic spinel structure with *a* = 8.13 Å. This unit cell parameter is slight larger than the cell dimension of Co_3_O_4_.

Fig. 3c shows an HRTEM image from the shell of a yolk–shell microsphere in Mn(1.5-24). The 2-dimensional lattice fringes allow us to measure the two *d*-spacings and their interplane angle more accurately, *d*_E_ = 4.81 Å, *d*_F_ = 2.98 Å and *α* = 86.5°. These values can fit a tetragonal unit cell with *a* = 5.77 Å and *c* = 9.00 Å by the indexing E: (101) and F: (11̄2̄) planes. Fig. 3d is a HRTEM image from a solid core of a yolk–shell microsphere. The measured *d*-spacings of *d*_G_ = 2.51 Å and *d*_H_ = 2.64 Å with the interplane angle of 37° can also be indexed to the G: (013) and H: (103) planes of the above unit cell. The similarity of the unit cells in the shell and the core implies that the structural difference is not the main reason of their separation.

Fig. 3e is an HRTEM image of the outer surface of a shell of a hollow yolk–shell microsphere in Mn(2-24). In the inset, the arrow in the TEM image indicates the gap between the core and the shell. Therefore, a thickness of the shell can be measured to be 60 to 120 nm. Two *d*-spacings are measured from a nanocrystal with a clearly 2D lattice, *d*_I_ = 2.64 Å and *d*_J_ = 2.46 Å, which can be indexed to the (103) and (2̄02) planes of the tetragonal unit cell of Mn_2_CoO_4_. A crystal with 2D fringes was also recorded from a core of a hollow yolk–shell microsphere in Mn(2-24) (Fig. 3f). The *d*-spacings are measured to be *d*_K_ = *d*_L_ = 4.82 Å with the interplane angle of 63°. These fringes can be indexed to the (011) and (011̄) planes of the unit cell of Mn_2_CoO_4_. Again, we expect the structural difference between the shell and the core is not significant.

The tetragonal distortion with Mn-doping from the cubic spinel Co_3_O_4_ leads to a simple relation of the unit cell axes between Co_3_O_4_ and Mn_2_CoO_4_, 
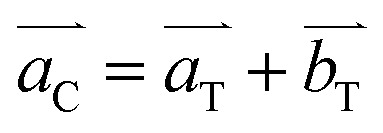
, 
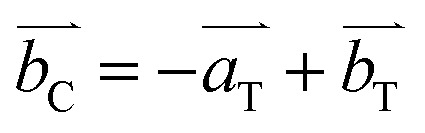
, and 
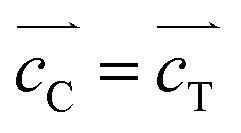
 (Fig. S4, ESI†). The extension is small along *a* and *b* axes, but is significant along the *c* axis due to a Jahn–Teller effect. Other compositions can be regarded as some intermediate phases between these two end compounds.

Energy dispersive X-ray spectroscopy (EDX) makes it possible to detect the local compositions of the samples (Table 1). It was found that, at a nanoscale, the samples are not homogeneous. The Mn/Co ratio in the shell-free microspheres in Mn(1-24) is 0.41, which is slightly lower than the nominal ratio of 0.5. Some shell-free microspheres were also observed in Mn(1.5-24). The EDX spectra from these particles show Mn/Co ratio of 0.70. The main phase in Mn(1.5-24) is solid yolk–shell microspheres, from which, the Mn/Co ratios are 1.08 in the shells and 0.84 in the cores. Some solid yolk–shell particles were also detected in Mn(2-24). The Mn/Co ratios are 2.01 in the shells and 0.92 in the cores in these particles. For the hollow yolk–shell particles in Mn(2-24), the Mn/Co ratios are 1.78 in the shells and 2.6 in the cores. In summary, the hollow cores possess the highest content of manganese (Mn/Co > 2); the shells of both yolk–shell spheres with either solid cores or hollow cores found in Mn(2-24) have a Mn/Co ratio *ca.* 2, while the shells found in Mn(1.5-24) have a lower Mn/Co ratio *ca.* 1, which is higher than the ratio in the solid cores; and the Mn/Co ratio in the shell-free microspheres is <0.8. In other words, the formation of the cores, either solid or hollow cores, in yolk–shell microspheres is related to high concentrations of Mn. The core–shell spheres were not observed in Mn(1-24), which contains a low content of Mn. This agrees with the results obtained by Fu *et al.*^20^ Some typical EDX spectra are presented in Fig. S5, ESI.† The different Mn/Co ratio between the core and the shell can be maintained since ionic diffusion across the gap is difficult.

**Table 1:** The Mn/Co ratios from the EDX results of the specimens. Types of spheres, I: shell-free spheres; II: solid core yolk–shell spheres; III: hollow core yolk–shell spheres

Sample	Location	Mn/Co	Sample	Location	Mn/Co
Mn(1-24)	I	0.41 ± 0.04	Mn(2-0.5)	Nanoparticle	1.38 ± 0.18
Mn(1.5-24)	I	0.70 ± 0.06		Nanosheet	5.1 ± 1.2
	Shell, II	1.08 ± 0.12	Mn(2-1)	Nanosheet	1.96 ± 0.16
	Core, II	0.84 ± 0.11		I	2.4 ± 0.9
Mn(2-24)	Shell, II	2.01 ± 0.02	Mn(2-2)	I	0.68 ± 0.03
	Core, II	0.92 ± 0.06		Shell, II	1.8 ± 0.3
	Shell, III	1.78 ± 0.16		Core, II	0.80 ± 0.02
	Core, III	2.6 ± 0.3		Shell, III	1.9 ± 0.3
Mn(1-0.5)	Nanoparticle	0.22 ± 0.02		Core, III	2.84 ± 0.07
Mn(1-1)	I	0.66 ± 0.11			

### Structures of specimens at early stages

Nevertheless, an investigation of the microstructures of the specimens at early stages of the crystal growth is probably helpful in understanding the formation mechanisms of the yolk–shell microspheres.

**Fig. 4:**
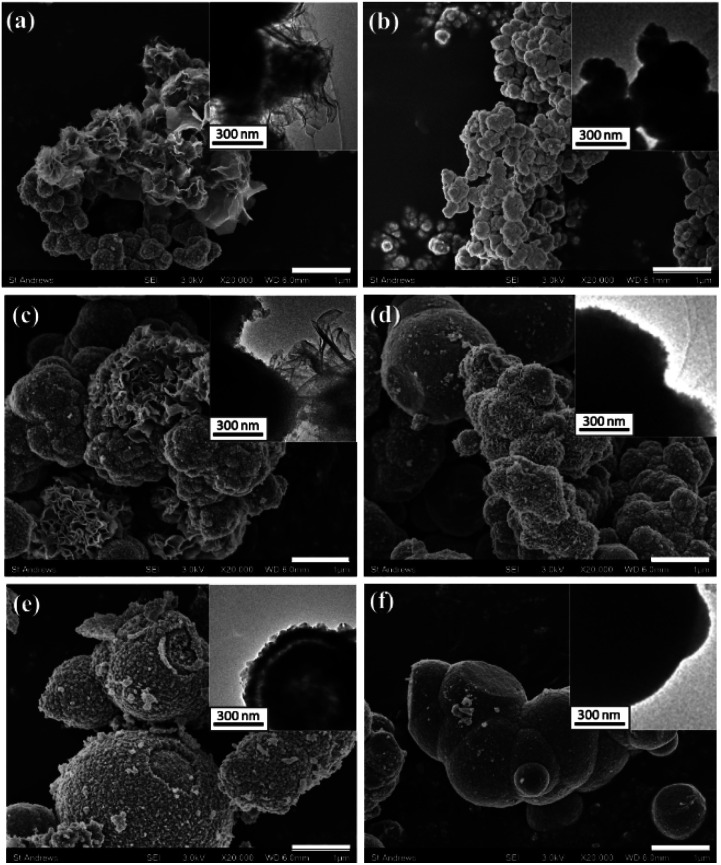
SEM images displaying morphology changes based on the variation of reaction time. Images were recorded from (a) Mn(2-0.5), (b) Mn(1-0.5), (c) Mn(2-1), (d) less developed particles in Mn(1-1), (e) Mn(2-2) and (f) Mn(1-1). The scale bars are 1 μm. The insets are the corresponding TEM images.

Two series samples of Mn(2-*t*) and Mn(1-*t*) with different reaction time, *t*, were produced. With 30 min reaction time, both samples of Mn(2-0.5) and Mn(1-0.5) contain only small particles of 200 nm to 300 nm in diameter, as shown in SEM and TEM images (Fig. 4a and b). However, there are a large number of very thin nanosheets in Mn(2-0.5), which is not observed in Mn(1-0.5). These nanosheets interlace each other and deposit on the surface of irregularly shaped small particles. The small particles are relatively Co-rich, having a Mn/Co ratio of 1.38 in Mn(2-0.5) and 0.22 in Mn(1-0.5). The nanosheets, on the other hand, have a much higher content of Mn with Mn/Co of 5.1 in Mn(2-0.5) (Table 1, Fig. S6, ESI†).

When the reaction time was elongated to 1 h, the irregular small particles and nanosheets further aggregated into larger particles, 1 μm or larger in diameter, in Mn(2-1) (Fig. 4c). At this stage, spherical morphology is still not yet observed. In Mn(1-1), on the other hand, small particles aggregated into large spheres, some with a rougher surface as shown in Fig. 4d, while others with a denser surface as shown in Fig. 4f. The involvement of the nanosheets in Mn(2-1) led to less dense particles and decelerated formation of spherical particles. According to the EDX results, the Mn/Co of the nanosheets in Mn(2-1) reduces from 5.1 of Mn(2-0.5) to 1.96 and that of irregular particles increases from 1.38 of Mn(2-0.5) to 2.4. The Mn/Co ratio of the spheres in Mn(1-1) also increases from 0.22 of small particles in Mn(1-0.5) to 0.66 (Fig. S6c, d and j, ESI†).

After reaction for 2 h, yolk–shell spheres appeared in Mn(2-2) specimen (Fig. 4e). The nanosheets were no longer observed. This yolk–shell structure did not develop in the Co-rich samples even the reaction time was elongated up to 24 h. Both yolk–shell structures with either a solid core or a hollow core were observed in Mn(2-2). The EDX results revealed the ratios of Mn/Co in the hollow-core particles to be 2.84 in the cores and 1.9 in the shells. The Mn/Co ratios in the solid-core particles were detected to be 0.80 in the core and 1.8 in the shells.

The phase evolution during the growth of the microspheres was studied using PXRD and HRTEM. From the PXRD patterns shown in Fig. S7, ESI†, it can be seen that the cobalt-rich samples did not undergo a phase transformation when the reaction time was increasing. Both PXRD patterns of Mn(1-1) and Mn(1-24) can be indexed to a cubic spinel structure. However, this is not the case for the manganese-rich samples. With a short reaction time up to 1 h, the PXRD pattern of Mn(2-1) can be indexed to a cubic spinel structure, although its crystallinity is poor. Bearing in mind that there are Mn-rich thin nanosheets in the samples, another crystalline phase could be Mn(OH)_2_-type, which is hexagonal, space group *P*3̄*m*1, with the unit cell parameters of *a* = 3.347 Å and *c* = 4.689 Å (JCPDS reference code: 01-073-8392). It is interesting to see that the diffraction peak positions of the cubic Mn_*x*_Co_3−*x*_O_4_ and hexagonal Mn(OH)_2_ are almost overlapped (see Fig. S7, ESI†). More likely Mn(2-1) contains two components, Co-rich cubic spinel irregular particles and Mn(OH)_2_-based Mn-rich nanosheets. With a further increase of the reaction time, the PXRD pattern of Mn(2-2) is obviously a tetragonal distorted spinel structure. The crystallinity is also greatly improved (Fig. S7, ESI†).

**Fig. 5:**
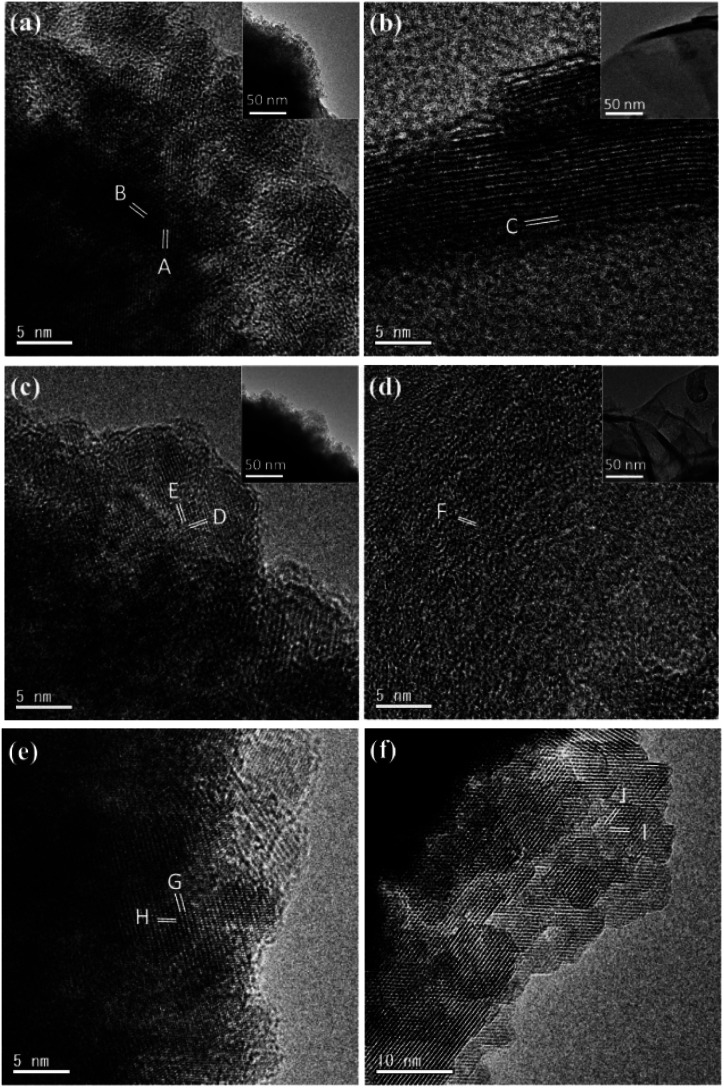
HRTEM images of specimens with short reaction times. (a) An irregular particle and (b) nanosheets in Mn(2-0.5). The marked fringes with *d*_A_ = 3.03 and *d*_B_ = 2.84 Å can be indexed to the (112) and (02̄0) planes in a tetragonal spinel structure. The fringes with *d*_C_ = 4.58 Å can be indexed to (001) of Mn(OH)_2_. (c) An irregular particle and (d) nanosheets in Mn(2-1). The marked fringes with *d*_D_ = 2.42 and *d*_E_ = 2.95 Å are indexed to the (022) and (200) planes of a tetragonal spinel structure. The *d*-spacing *d*_F_ = 2.41 Å, matches the (011) planes of Mn(OH)_2_. (e) Crystallites in Mn(1-0.5). The marked fringes, *d*_G_ = 4.04, *d*_H_ = 2.50 Å, are indexed to the (020) and (311) planes of a pseudo-cubic spinel structure. (f) Crystallites in Mn(1-1). The marked fringes, *d*_I_ = 4.50, *d*_J_ = 4.72 Å, are indexed to the (002) and (111) planes of a pseudo-cubic spinel structure.

HRTEM images of the samples reveal more details of the crystalline phases. Fig. 5a shows a HRTEM image from an irregular particle in Mn(2-0.5). Many very small nanocrystallites, ∼5 nm in diameter, are embedded in an amorphous phase. The measured *d*-spacings, 3.03 and 2.84 Å, cannot be indexed to a cubic unit cell, but can be indexed to the (112) and (02̄0) planes of a tetragonal spinel structure. Fig. 5b is a HRTEM image from a nanosheet in the same sample with a profile view, which shows a layered structure with an interlayer spacing of 4.58 Å, corresponding to *d*_(001)_ of Mn(OH)_2_. A model of the layered structure of Mn(OH)_2_ is shown in Fig. S8, ESI†. The nanosheets have much higher crystallinity than the irregular particles.

The HRTEM images in Fig. 5c and d were recorded from Mn(2-1). The nanocrystallites in the irregular shaped particles seem to be larger than those in Mn(1-0.5) and the proportion of the amorphous phase significantly reduced. The fringes can also be indexed to a pseudo-cubic unit cell. The fringes *d*_D_ = 2.42 and *d*_E_ = 2.95 Å can be indexed to the (022) and (200) planes of a tetragonal spinel structure. The image viewed down the face of the nanosheets shows a domain structure probably after damage of the Mn(OH)_2_-type structure. The fringes with *d*_F_ = 2.41 Å, matching to the *d*-spacing of (011) of Mn(OH)_2_-type hexagonal structure (Fig. 5d).

In the Co-rich system Mn(1-0.5), no nanosheets were detected. The irregular shaped particles contain nanocrystallites with less amount of disordered phase. Measurement of the *d*-spacings and interplane angles allow us to find that the crystallites are not perfect cubic, but possess a tiny distortion due to Mn-doping in Co_3_O_4_ (Fig. 5e). In Mn(1-1), at least in the surface area, there is much less disordered phase and most nanocrystallites increase their crystallinity and even self-orientated (Fig. 5f).

### Proposed formation mechanisms of the microstructures

**Fig. 6:**
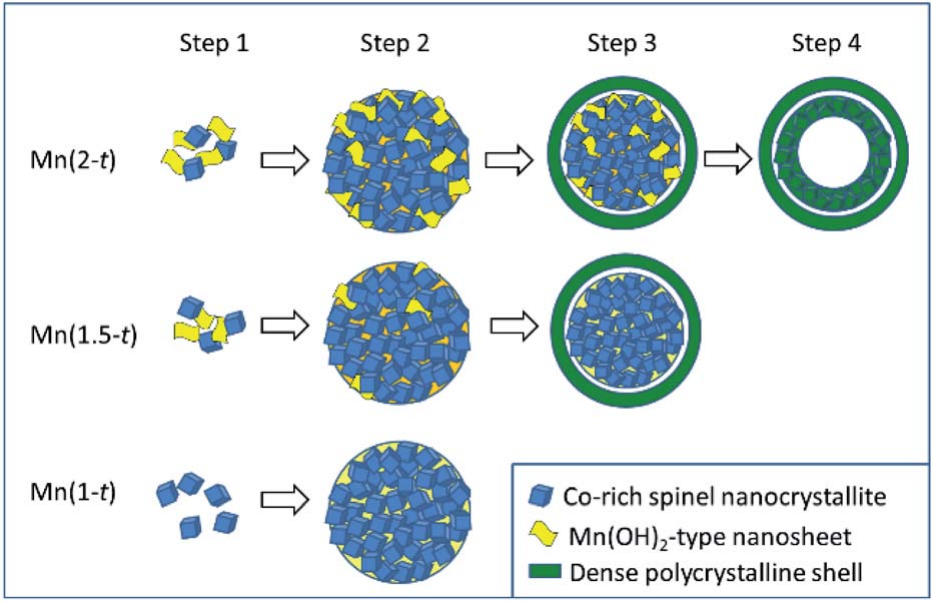
Schematic diagram of the proposed formation mechanisms of hollow yolk–shell microspheres, solid yolk–shell microspheres of Mn-rich Mn_*x*_Co_3−*x*_O_4_.

The chemical reactions behind the growth of spinel Mn_*x*_Co_3−*x*_O_4_ have been discussed in detail by Fu *et al.*^20^ Redox reactions in the solvothermal synthetic system would generate reduced gas products, NO and N_2_O, as well as OH^−^ anions. Mn^2+^ and Co^2+^ cations initially form (Mn, Co)(OH)_2_ or (Mn, Co)_3_(OOH)_3_ precipitates. The metal cations in the latter are partially oxidised from 2+ to 3+. Both hydroxide and oxide hydroxide transfer into spinel oxide *via* combination with more ions and dehydration. The obvious new phenomena observed in our work were that Co-doped Mn(OH)_2_ nanosheet was an important component in the polycrystalline microspheres, leading to the formation of the yolk–shell structures and the ratio of Mn/Co in the nanoparticles and nanosheets varied with the reaction time. Based on these observations, we are able to propose formation mechanisms of the microspheres in several steps (Fig. 6).

Step 1. In all the solutions, Co^2+^ were partially oxidised and formed Co-rich spinel nanocrystallites, <5 nm in diameter. In a Mn-poor system, Mn(1-0.5), the Mn cations substituted Co in Co_3_O_4_ to form pseudo-cubic spinel nanocrystallites. No Mn(OH)_2_-type nanosheets formed. In a Mn-rich system, Mn(2-0.5) or Mn(1.5-0.5), in addition of the formation of the Mn-doped Co_3_O_4_ nanocrystallites, most Mn^2+^ cations form Mn(OH)_2_-type nanosheets with Co-doping. Solid solution of Mn_*x*_Co_3−*x*_O_4_ could form at least in a range from Co_3_O_4_ to Mn_2_CoO_4_. However, at a relatively low temperature, diffusion of ions was very slow.

Step 2. The spinel nanocrystallites and Mn(OH)_2_-type nanosheets self-assembled into polycrystalline particles due to strong inter-particle interactions and gradually increased their size to form microspheres as observed in Mn(2-1) and Mn(1.5-1). During this process, ion diffusion would take place between the Co-rich nanocrystallites and Mn-rich nanosheets. Decomposition of the Mn_1−*x*_Co_*x*_(OH)_2_ generated a non-crystalline phase. Therefore, the microspheres contain nanosheets and nanocrystallites embedded in the disordered substrate. Obviously, Mn(OH)_2_-type nanosheets included in the aggregates greatly reduced the density of the microspheres. On the other hand, similar to Mn(1-0.5), large Mn(OH)_2_-type nanosheets did not appear in Mn(1-1) due to a low concentration of Mn. The Co-rich nanocrystallites aggregated into some denser microspheres. At this stage, we saw that, unlike Co-free Mn(3-24) in which large octahedral single crystals of Mn_2_O_3_ formed *via* the classical growth route, in the Co-containing systems, Mn_*x*_Co_3−*x*_O_4_ nanocrystallites formed. Before these nanocrystallites grew up as free crystals, they aggregated into large spheres. The further crystal growth inside such microspheres was largely inhibited.

Step 3. The microspheres, consisting of Co-rich nanocrystallites, Mn-rich nanosheets and amorphous substrate would inevitably undergo a process of shrinkage *via* Ostwald ripening. The surface of the spherical particles became the most active site for re-crystallisation with less restrictions of space and mass transportation. In many cases, such a surface re-crystallisation would lead to formation of single-crystal polyhedral shells, followed by extension of crystal growth from the surface to the core, in the so-called reversed crystal growth route.^26^ A very interesting example was demonstrated by Yang *et al.*^27^ They solvothermally synthesised CaTiO_3_ nanocubes, which self-assembled into microspheres. If no water was added, the surface of these spherical particles would be walnut-like without re-crystallisation into single crystal shells. When a small amount of water (1.25% or 5%) was added in the system, the surface of the microspheres re-crystallised to single crystal cubic shells. The crystallisation extended from the surface to the core, leading to a final morphology of hollow cubes. Similarly, the polycrystalline microspheres in ethanol in the present work also underwent surface re-crystallisation to increase the crystallinity, although a single-crystal shell was not resulted. All the nanocrystallites in the surface area connected each other to form a rigid polycrystalline shell. During the dehydration of the Mn(OH)_2_-type nanosheets and re-crystallisation, shrinkage inevitably occurred. If the shrinkage was from the centre to the surface of a polycrystalline microsphere, the size of the microsphere would be reduced. If this process went to an opposite direction, from the surface to the centre, a rigid shell would form first and its size was unchanged. Further shrinkage would generate cavities inside the particle. This was the reason for the formation of the gap between the shell and the core in Mn(2-2) and Mn(1.5-2), forming yolk–shell microspheres. In Mn(1-24), the density of the microspheres was high without Mn(OH)_2_-type nanosheets. The change of the volume during the shrinkage was not so significant. Therefore, no separated shells would develop.

Step 4. With further reaction, the cores with a high Mn content of yolk–shell microspheres in Mn(2-2) would undergo a similar reversed shrinkage to generate an empty centre. The condition allowing this to happen was a high proportion of Mn(OH)_2_-type nanosheets or a low density of metal cations in the cores. The EDX results of high Mn content in hollow cores and low content in solid cores support this argument. Consequently, the morphology evolution of these particles is mainly influenced by the content of Mn(OH)_2_-type nanosheets in the aggregates, therefore by the original concentration of Mn.

## Experimental

### Sample preparation

The synthesis method used in the present work was similar to that reported by Fu *et al.*^20^ with some minor modifications. In a typical procedure, stoichiometric amounts of manganese(ii) nitrate tetrahydrate and cobalt(ii) nitrate hexahydrate (both from Sigma Aldrich), 3 mmol in total (Mn : Co = 3 : 0, 2 : 1, 1 : 2 or 0 : 3), were added into 15 ml of absolute ethanol and stirred by a magnetic stir bar for 30 min. The solution was then transferred into a 30 ml Teflon liner autoclave and heated in an oven at 140 °C for a time from 0.5 to 24 h. After the heating process, the autoclave was allowed to cool to room temperature naturally. The precipitate was then washed with ethanol and distilled water several times and dried in an oven at 60 °C.

### Specimen characterisation

Initial specimen characterisation was performed using PXRD on a PANalytical Empyrean diffractometer, using Cu K_α_ radiation. Particle size and morphology of the samples were examined using SEM images, which were recorded on a JEOL JSM-6700F field emission gun microscope with an accelerating voltage of 3 or 5 kV. The samples for SEM was coated with gold using a Quorum Technologies Q150RES sputter to reduce the charging effect. The local crystal structures were detected using HRTEM. The TEM and HRTEM images were recorded on a JEOL JEM-2011 electron microscope operating at 200 kV. The local Mn : Co ratios of the samples were detected by using EDX. For some specimens with a poor homogeneity, more than 10 particles were examined to get an average value of Mn/Co.

## Conclusions

The formation of yolk–shell microspheres of spinel Mn_*x*_Co_3−*x*_O_4_ relies on co-aggregation of Mn-doped Co_3_O_4_ nanocrystallites and Co-doped Mn(OH)_2_-type nanosheets to form low-density polycrystalline microspheres, followed by a reversed shrinkage, a similar process of previously reported reversed crystal growth route. Even in the same sample of Mn(2-2), all three microspheres, shell-free spheres, solid core and hollow core yolk–shell microspheres, formed as governed by the proportion of the Mn(OH)_2_-type nanosheets in the precursor microspheres. It was noted from the EDX results (Table 1), when the Mn/Co ratio was low (∼0.7), only solid spheres formed. The shells of the two types of yolk–shell microspheres had a Mn/Co ratio close to the nominal value (∼2). The Mn/Co ratio of the solid cores was also low (<1) and the value was high (>2.5) in the hollow cores. Consequently, in this synthetic system, changing the Mn/Co ratio can control the products of shell-free, solid core yolk–shell and hollow core yolk–shell microspheres. The produced polycrystalline shells are rigid enough to achieve a high stability and are porous, having wider potential applications in comparison with hollow single crystals. The present work sheds light on understanding formation mechanisms and controlled fabrication of core–shell and yolk–shell microspheres.

## Conflicts of interest

There are no conflicts to declare.

## Supplementary Material

RA-011-D1RA04996H-s001
