# Supplementary material for: Formation mechanism of MnxCo3−xO4 yolk–shell structures
Source: RSC Adv. 2021 Sep 1;11(47):29108–14. doi: 10.1039/d1ra04996h (PMC9040891; doi:10.1039/d1ra04996h)
Supplement: RA-011-D1RA04996H-s001 [file RA-011-D1RA04996H-s001.pdf]

Electronic Supplementary Material (ESI) for RSC Advances.
This journal is © The Royal Society of Chemistry 2021

Supporting Information

Formation Mechanism of $Mn_xCo_{3-x}O_4$ Yolk-shell Structures

Chang-Yang Chiang and Wuzong Zhou

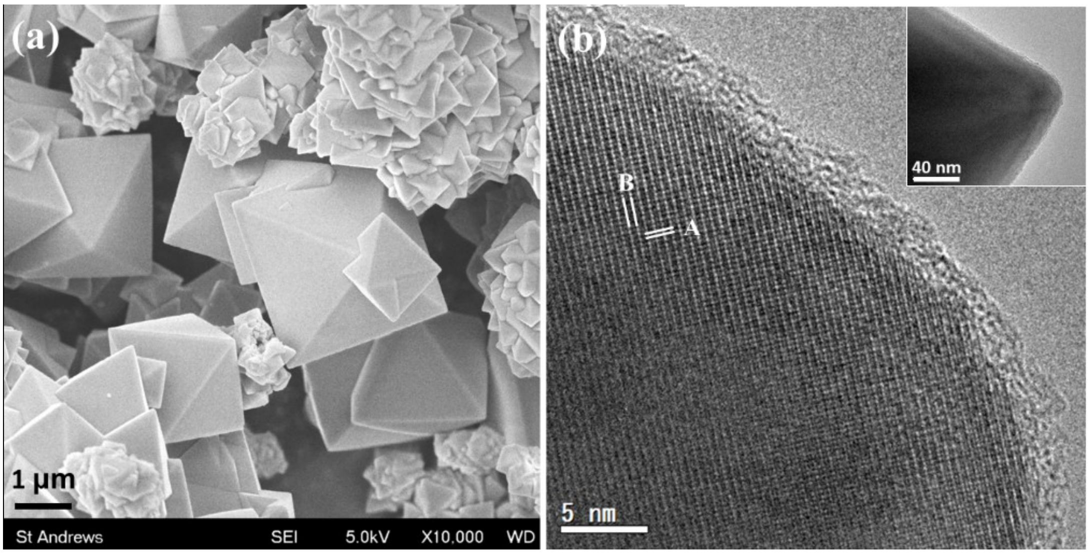

Fig. S1 (a) SEM image of specimen Mn(3-24), showing octahedral crystals of Mn_2O_3. (b) HRTEM image of an octahedral particle with the lattice fringes being indexed to the cubic Mn_2O_3 unit cell, $d_A = d_{200} = 4.75Å$ and $d_B = d_{013} = 2.96Å$ with the interplane angle of 90°. The inset is the corresponding TEM image at a low magnification.

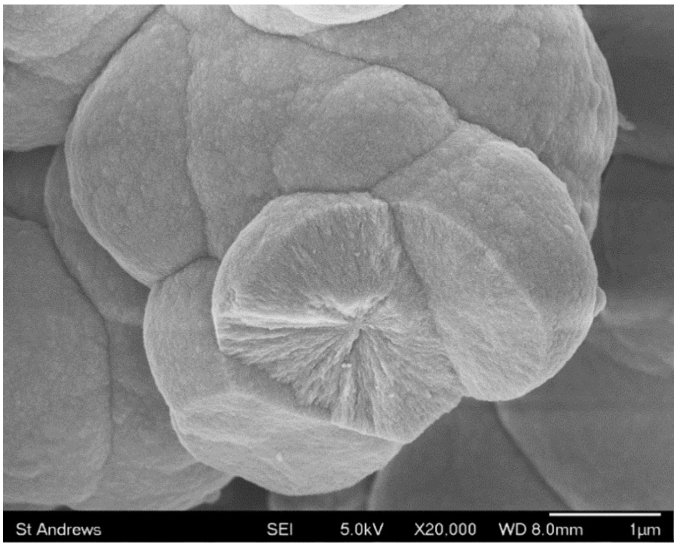

Fig. S2 High resolution SEM image of Mn(0-24). The broken spheres show the dense and polycrystalline cores.

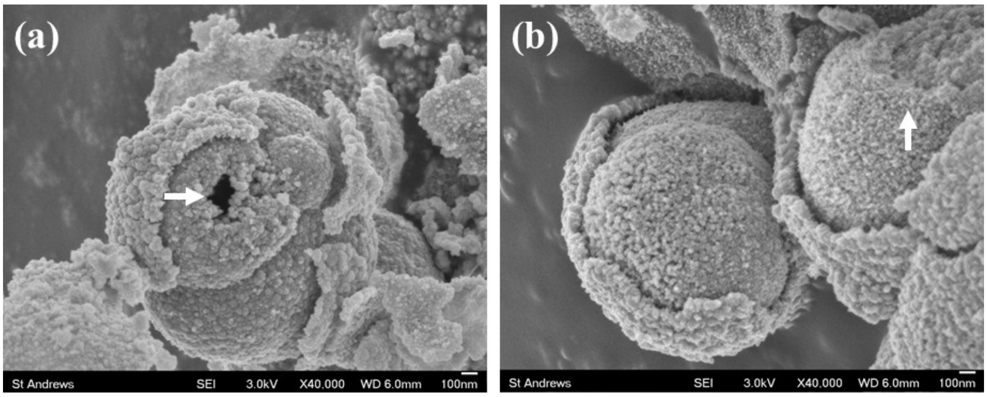

Fig. S3 High resolution SEM images recorded from Mn(2-24). The arrows indicate (a) hollow and (b) solid cores.

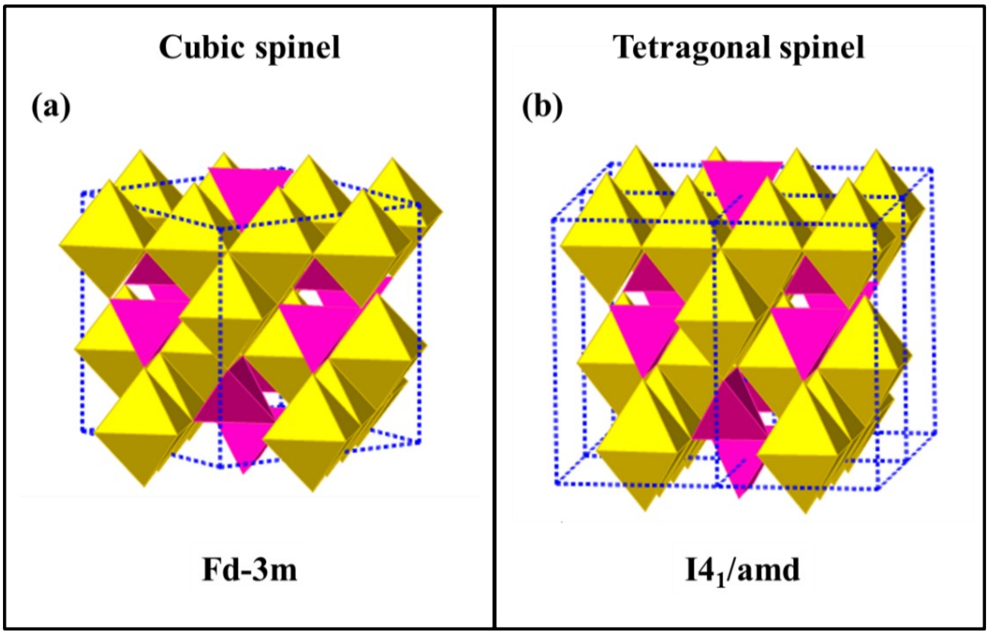

Fig. S4 A diagram showing the relationship between (a) the cubic and (b) the tetragonal spinel structures via the polyhedral models. The unit cells are represented by the dotted lines.

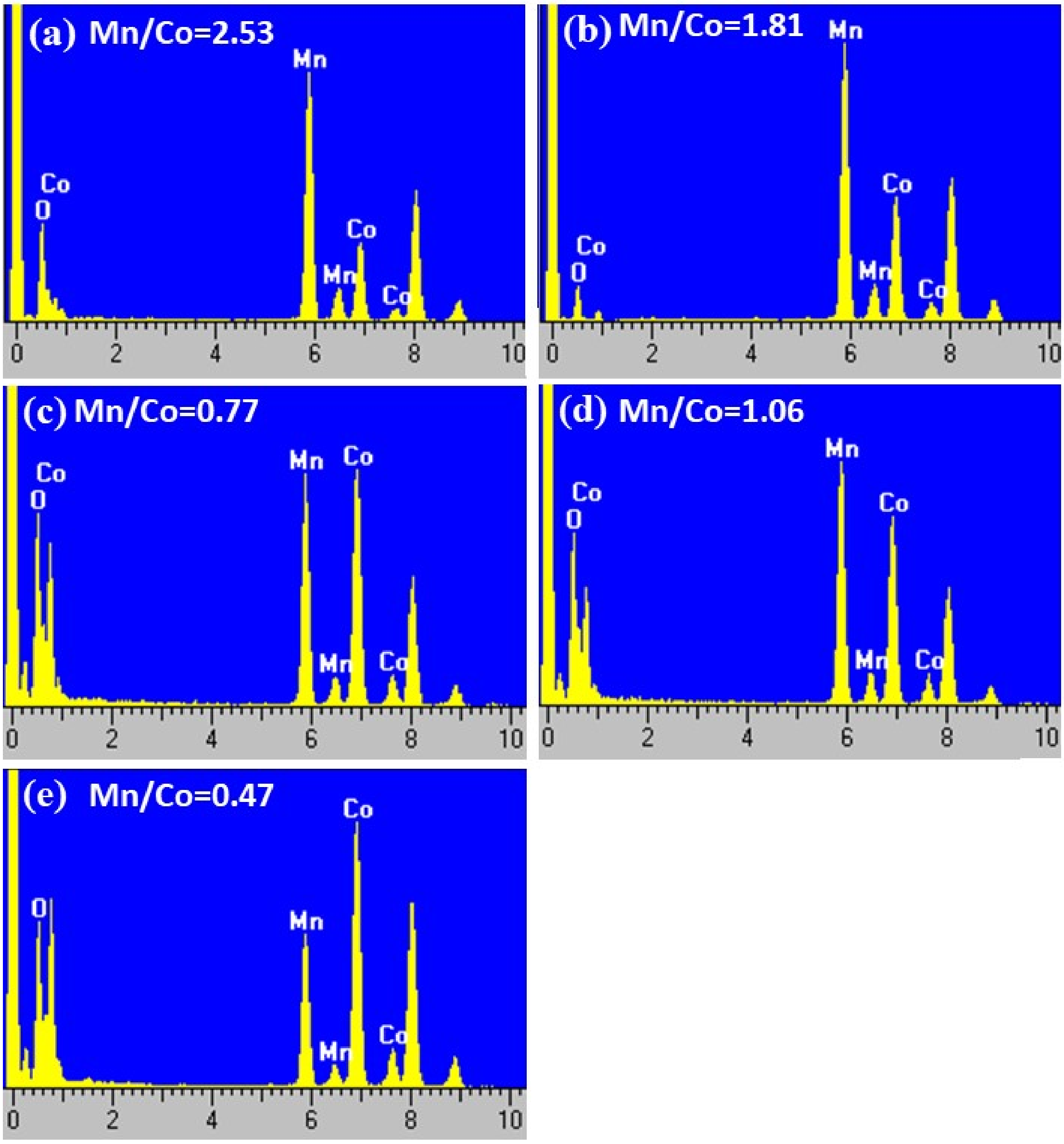

Fig. S5 Some typical EDX spectra from (a) a core and (b) a shell of a hollow yolk-shell microsphere in Mn(2-24), (c) a core and (d) a shell of a solid yolk-shell microsphere in Mn(1.5-24), (e) a shell-free microsphere in Mn(1-24).

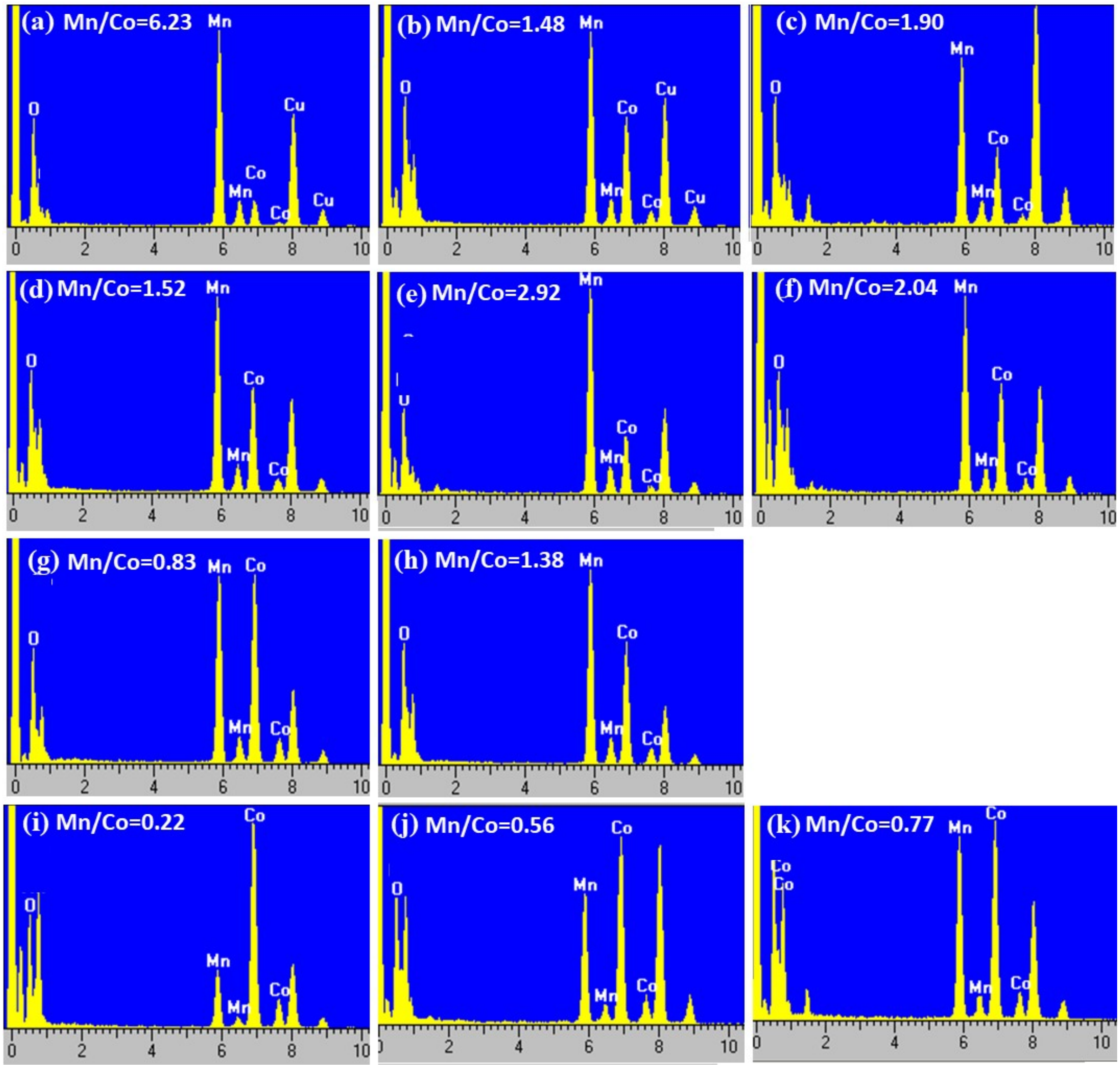

Fig. S6 EDX spectra from specimens at early stages of the crystal growth. (a) Nanosheets and (b) small particles in Mn(2-0.5). (c) Nanosheets and (d) growing particles in Mn(2-1). (e) Core and (f) shell of hollow yolk-shell spheres in Mn(2-2). (g) Core and (h) shell of solid yolk-shell spheres in Mn(2-2). (i) Small particles in Mn(1-0.5). (j) Medium sized particles and (k) large particles in Mn(1-1).

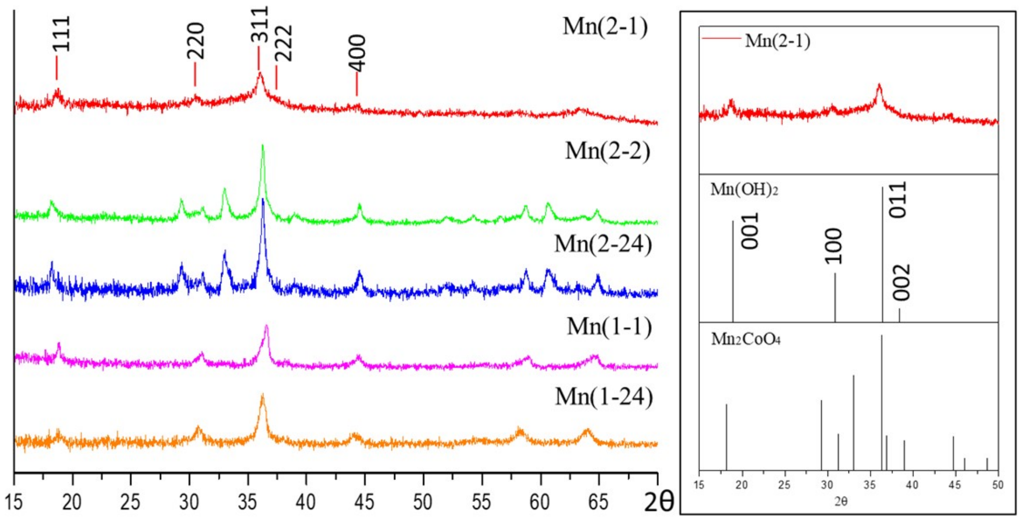

Mn(OH)$_2$			$Mn_xCo_{3-x}O_4$	
hkl	2θ	d-spacing	*hkl*	d-spacing
001	18.7	4.689	111	4.694
100	31.1	2.899	220	2.874
011	36.5	2.466	311	2.451
002	38.0	2.345	222	2.347
			400	2.033

Fig. S7 XRD patterns of samples with short reaction times. The pattern of Mn(2-1) is indexed to a cubic spinel unit cell. The right picture shows a comparison of the pattern Mn(2-1) with the standard pattern of hexagonal unit cell of $Mn(OH)_2$. The table shows a comparison of the *d*-spacings of hexagonal $Mn(OH)_2$ with $a = 3.347$ and $c = 4.689$ Å and cubic $Mn_xCo_{3-x}O_4$ with $a = 8.130$ Å.

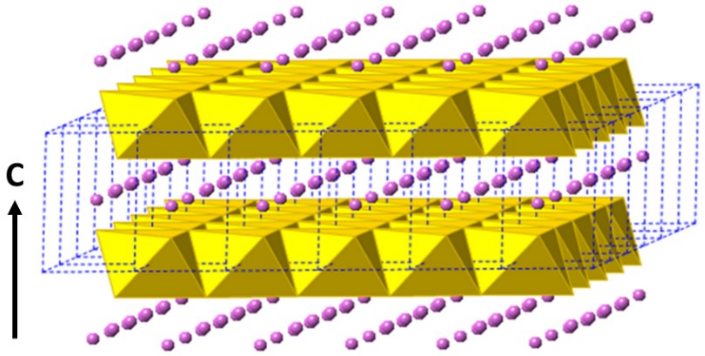

Fig. S8 The crystal structure of $Mn(OH)_2$ represented by the MnO_6 octahedra (yellow) and the hydrogen atom (pink). The layers, composed of edge-sharing octahedra, can bind to each other by the Van der Waals forces.
